# Selenium-Containing Multi-Resonance Thermally Activated Delayed Fluorescence Host Material for Green and Red Phosphorescent OLEDs

**DOI:** 10.3390/ma18092040

**Published:** 2025-04-29

**Authors:** Hyukmin Kwon, Seokwoo Kang, Sangwook Park, Saeyoung Oh, Sang-Tae Kim, Kiho Lee, Hayoon Lee, Jongwook Park

**Affiliations:** Integrated Engineering, Department of Chemical Engineering, Kyung Hee University, Yongin-si 17104, Republic of Korea; hm531@khu.ac.kr (H.K.); swkang@khu.ac.kr (S.K.); pswook@khu.ac.kr (S.P.); tpdud5821@khu.ac.kr (S.O.); kimst2@khu.ac.kr (S.-T.K.); kiholee@khu.ac.kr (K.L.); kssarang1@khu.ac.kr (H.L.)

**Keywords:** organic light-emitting diodes, multi-resonance thermally activated delayed fluorescence, phosphorescence, host material

## Abstract

We report the molecular design and synthesis of a novel selenium-containing multi-resonance thermally activated delayed fluorescence (MR-TADF) host material, 3,6-di-tert-butyl-9,16-dioxa-15-selena-4b-boraindeno[2,1-a]naphtho[3,2,1-de]anthracene (TDBA-SePh), for green and red phosphorescent organic light-emitting diodes (PhOLEDs). By incorporating selenium into the DOBNA-based MR-TADF backbone, the reverse intersystem crossing (RISC) process was effectively activated, leading to enhanced utilization of triplet excitons. The corresponding RISC rate was determined to be 3.91 × 10^4^ s^−1^. When applied to PhOLED devices, TDBA-SePh-based green and red OLEDs exhibited higher external quantum efficiency (EQE) and reduced efficiency roll-off compared to conventional mCP-based host materials. At a luminance of 1000 cd m^−2^, the green and red devices exhibited roll-off values of 2.5% and 4.3%, respectively. This improvement is attributed to the incorporation of selenium as a heteroatom, which accelerates the RISC process, thereby suppressing triplet-triplet annihilation (TTA). These results suggest that adopting a similar molecular design strategy can not only reduce efficiency roll-off but also enhance device efficiency and operational stability, offering significant potential for future OLED applications.

## 1. Introduction

Organic light-emitting diodes (OLEDs) have attracted significant attention from both academia and industry due to their high contrast ratio, self-emissive properties, flexibility in display applications, and low energy consumption [1,2,3,4,5,6]. Compared to fluorescent OLEDs, phosphorescent OLEDs (PhOLEDs) can utilize both singlet (25%) and triplet (75%) excitons, theoretically enabling an internal quantum efficiency (IQE) of 100% [7,8,9]. However, the long exciton lifetime of phosphorescence can cause efficiency degradation in devices due to concentration quenching and triplet-triplet annihilation (TTA) [10,11,12,13]. To address these challenges, PhOLEDs commonly use a host-dopant approach, which reduces the accumulation of triplet excitons, thereby improving both the luminescence efficiency and device stability [14,15,16]. Therefore, the design and development of suitable host materials are essential for improving the efficiency of PhOLEDs. In 2016, Hatakeyama et al. designed a multi-resonance thermally activated delayed fluorescence (MR-TADF) molecule by utilizing the opposing electronic effects of nitrogen (N) and boron (B) atoms. Their study demonstrated that this approach enables atomic-level control over the distribution of the highest occupied molecular orbital (HOMO) and lowest unoccupied molecular orbital (LUMO) [17,18]. This molecular design enabled a small singlet-triplet energy gap (ΔE_ST_), high oscillator strength, and excellent luminescence efficiency. Due to its rigid polycyclic aromatic structure, it exhibited a narrow emission spectrum with a full width at half maximum (FWHM) of less than 30 nm. These attributes make MR-TADF emitters promising candidates for high-efficiency OLED applications, demonstrating over 20% external quantum efficiency (EQE) and superior electroluminescence (EL) color purity. However, MR-TADF molecules generally possess delayed fluorescence lifetimes on the order of several tens of microseconds, which significantly contributes to efficiency roll-off under high current density conditions. This is primarily due to the increased occurrence of triplet exciton quenching processes, such as TTA and singlet-triplet annihilation (STA) [19,20,21]. Furthermore, several studies have proposed the incorporation of heavy atoms into the molecular structure of MR-TADF compounds to enhance spin-orbit coupling (SOC) and consequently improve the rate of reverse intersystem crossing (RISC) [22,23,24]. Selenium (Se)-containing compounds typically exhibit distinct chemical and electronic properties due to their pronounced heavy atom effect. In particular, the incorporation of selenium into MR-TADF systems has been found to significantly enhance SOC and accelerate the RISC process through the external heavy-atom effect (EHAE) [25]. This enhancement effectively suppresses efficiency roll-off and leads to a notable improvement in device performance. Yasuda et al. developed a selenium-containing MR organoboron blue dopant (CzBSe), achieving a high RISC rate. OLEDs utilizing this emitter exhibited narrow blue emission characteristics due to the MR effect, along with a significant reduction in efficiency roll-off. As a result, the devices demonstrated outstanding EL performance, achieving an EQE of 23.9% [26]. Wong et al. reported that the MR-TADF emitter SetBuNBN, developed by incorporating peripheral Se atoms, exhibited a fast RISC rate of 2.30 × 10^5^ s^−1^. The corresponding OLED demonstrated excellent efficiency roll-off characteristics, maintaining an EQE of 24.4% at 100 cd m^−2^ and 19.7% at 1000 cd m^−2^ [27]. Shi-Jian et al. reported the blue MR-TADF emitters sSeDDBN and PsSe-DABNA, which incorporate a selenium-containing spiro donor moiety. sSeDDBN exhibited a high RISC rate of 2.73 × 10^5^ s^−1^, and OLEDs utilizing this emitter demonstrated excellent EL performance, achieving a maximum EQE of 28.5% [28].

However, research on MR-TADF host materials incorporating Se atoms has not yet been reported. This study presents the design, synthesis, and characterization of a DOBNA-derived boron-based n-type TADF host material, named TDBA-SePh (3,6-di-tert-butyl-9,16-dioxa-15-selena-4b-boraindeno[2,1-a]naphtho[3,2,1-de]anthracene).

The proposed MR-TADF host material features a structural design concept incorporating a Se atom, with a fused framework consisting of a DOBNA core and para-positioned phenyl groups. The incorporation of a Se atom in the synthesized material is expected to facilitate a high RISC rate. Furthermore, its TADF characteristics and excellent thermal stability suggest its potential as a promising novel phosphorescent host material. To evaluate the host performance of TDBA-SePh, phosphorescent green, and red doped devices will be fabricated and systematically analyzed.

## 2. Materials and Methods

### 2.1. Synthesis

#### 2.1.1. Synthesis of 1-Bromo-3,5-bis-(4-tert-butyl-phenoxy)-benzene (**1**)

1-Bromo-3,5-difluorobenzene (10.0 g, 51.8 mmol), 4-tert-Butylphenol (19.5 g, 129.5 mmol), and potassium carbonate (17.9 g, 129.5 mmol) were dissolved in anhydrous N-Methyl-2-pyrrolidone (NMP) (50 mL) under a nitrogen atmosphere. After stirring at 170 °C 20 h, the reaction mixture was cooled to room temperature, and the solution was extracted with dichloromethane and water three times. The organic phase was dried with anhydrous MgSO_4_ and filtered. The solvent was condensed by evaporation under a vacuum precipitated with methanol and subjected to vacuum filtration, obtaining a pure white solid (yield: 75%). ^1^H NMR (400 MHz, Chloroform-*d*) δ 7.39–7.31 (m, 4H), 6.99–6.90 (m, 4H), 6.78 (d, J = 2.2 Hz, 2H), 6.56 (t, J = 2.2 Hz, 1H), 1.31 (s, 18H).

#### 2.1.2. Synthesis of 2-[3,5-Bis-(4-tert-butyl-phenoxy)-phenyl]-4,4,5,5-tetramethyl-[1,3,2]dioxaborolane (**2**)

In a 3-neck round flask, compound (**1**) (5.0 g, 11.0 mmol), potassium acetate (2.7 g, 27.6 mmol), Bis(pinacolato)diboron (3.1 g, 12.1 mmol), and 1,4-dioxane (60 mL) were combined under a nitrogen atmosphere. The mixture was stirred for 30 min. Pd(dppf)Cl2 (0.24 g, 0.3 mmol,) was added, and the temperature was set to 110 °C. The reaction was carried out for 6 h, with the reaction progress monitored intermittently by TLC. Extraction was performed using a mixture of dichloromethane (DCM) and deionized water (D.I water), followed by drying with MgSO_4_ to remove moisture. After maximum solvent removal, the obtained white solid was adsorbed and subjected to column chromatography (DCM: n-hexane = 1:9 *v*/*v*). The solvent was evaporated, yielding a white solid, which was then precipitated with methanol and subjected to vacuum filtration, obtaining a pure white solid (yield: 81%). ^1^H NMR (400 MHz, DMSO-*d*_6_) δ 7.41–7.33 (m, 4H), 6.97–6.91 (m, 4H), 6.86 (d, J = 2.4 Hz, 2H), 6.69 (t, J = 2.4 Hz, 1H), 1.24 (s, 18H), 1.20 (s, 12H).

#### 2.1.3. Synthesis of 2′-Bromo-3,5-bis-(4-tert-butyl-phenoxy)-biphenyl (**3**)

Compound (**2**) (1.0 g, 2.0 mmol), 1,2-dibromobenzene (0.28 mL, 2.4 mmol), potassium carbonate (0.69 g, 5.0 mmol), and tetrakis(triphenylphosphine)palladium(0) (0.09 g, 0.07 mmol) were dissolved in 12 mL of tetrahydrofuran (THF) and 4 mL of D.I water at room temperature under an argon atmosphere. After stirring at 80 °C 12 h, the reaction mixture was cooled to room temperature, and the solution was extracted with DCM and D.I water three times. The organic phase was dried with anhydrous MgSO_4_ and filtered. The solvent was condensed by evaporation under a vacuum and the crude was purified through the silica gel column chromatography (DCM: n-hexane = 1:19 *v*/*v*) to afford white solid (yield: 58%). ^1^H NMR (400 MHz, DMSO-*d*_6_) δ 7.66 (dd, J = 8.0, 1.2 Hz, 1H), 7.40–7.33 (m, 6H), 7.29–7.24 (m, 1H), 7.02–6.97 (m, 4H), 6.62 (d, J = 2.2 Hz, 2H), 6.51 (t, J = 2.2 Hz, 1H), 1.23 (s, 18H).

#### 2.1.4. Synthesis of (3′,5′-Bis(4-(tert-butyl)phenoxy)-[1,1′-biphenyl]-2-yl)boronic acid (**4**)

Compound (**3**) (4.0 g, 7.6 mmol) was dissolved in an anhydrous THF solution (40 mL) and stirred at −78 °C. Then, 2.0 M n-BuLi (4.2 mL, 8.3 mmol) was added. Triethyl borate (2.6 mL, 15.1 mmol) was added to the reaction after 30 min. After 12 h, the solution was acidified with 2 N HCl solution at room temperature and extracted with ethyl acetate and DI water. The organic layer was dried with anhydrous MgSO_4_ and filtered. The solvent was condensed by evaporation under a vacuum and the crude was purified through the silica gel column chromatography (ethyl acetate: n-hexane = 1:19 *v*/*v*). Reprecipitation of the residue from THF/n-hexane (1:4 vol ratio) to afford white solid (yield: 51%). ^1^H NMR (400 MHz, Chloroform-*d*) δ 7.39–7.32 (m, 8H), 7.01–6.97 (m, 5H), 6.70–7.68 (m, J = 1.7 Hz, 3H), 3.48–3.47 (d, 2H), 1.31 (s, 18H).

#### 2.1.5. Synthesis of 2,4-Bis-(4-tert-butyl-phenoxy)-dibenzoselenophene (**5**)

10 mL Schlenk tube equipped with a stir bar was charged with Compound (**4**) (1.0 g, 1.9 mmol), Se powder (0.4 g, 5.1 mmol), AgNO_2_ (0.06 g, 0.4 mmol) and K_2_S_2_O_8_ (0.7 g, 2.4 mmol). Dioxane (10 mL) was added to the Schlenk tube via a syringe through a rubber septum. The mixture was stirred at 130 °C in a preheated heating mantle for 24 h. After cooling to room temperature, the reaction solution was diluted with methylene chloride, filtered through celite, and concentrated under reduced pressure.

The solvent was condensed by evaporation under a vacuum and the crude was purified through the silica gel column chromatography (DCM: n-hexane = 1:9 *v*/*v*) to afford white solid (yield: 29%). ^1^H NMR (400 MHz, Chloroform-*d*) δ 7.95–7.88 (d, 1H), 7.82–7.75 (d, 1H), 7.63 (s, 1H), 7.39–7.35 (m, 4H), 7.33–7.29 (dd, 2H), 6.99–6.96 (m, 2H), 6.93–6.88 (dd, 2H), 1.33 (s, 9H), 1.30 (s, 9H).

#### 2.1.6. Synthesis of 3,6-di-tert-butyl-9,16-dioxa-15-selena-4b-boraindeno[2,1-a]naphtho[3,2,1-de]anthracene (TDBA-SePh)

Compound (**5**) (1.0 g, 1.9 mmol) was dissolved in anhydrous tert-Butylbenzene (12 mL) under an argon atmosphere. After stirring for 30 min, a solution of 1.7M t-BuLi (2.09 mL, 2.3 mmol) was added slowly to the solution at −30 °C. Then, the mixture was stirred at room temperature for 1 h. Then boron tribromide (0.22 mL, 2.3 mmol) was slowly added at −50 °C, and the reaction mixture was then maintained at room temperature for 30 min, followed by a 1 h reaction at 45 °C. Then N, N-diisopropylethylamine (0.62 mL, 3.6 mmol) was slowly added at −15 °C. The mixture was stirred at room temperature for 30 min, and 140 °C for 20 h. After completion of the reaction, the mixture was cooled to room temperature and extracted three times with dichloromethane (DCM) and deionized water. The organic layer was dried over anhydrous MgSO_4_ and filtered. The solvent was condensed by evaporation under a vacuum and the crude was purified through the silica gel column chromatography (DCM: n-hexane = 1:19 *v*/*v*) to afford yellow solid (yield: 18%). ^1^H NMR (400 MHz, Chloroform-*d*) δ 8.80–8.78 (d, 2H), 8.27–8.25 (d, 2H), 8.00–7.98 (d, 2H), 7.83–7.78 (s, 2H), 1.47 (s, 18H) ppm. HRMS(FAB+): calculated for C_32_H_29_BO_2_Se 536.1426, found: 536.1424.

## 3. Results and Discussion

### 3.1. Molecular Design, Synthesis, and Optical Properties

**Scheme 1:**
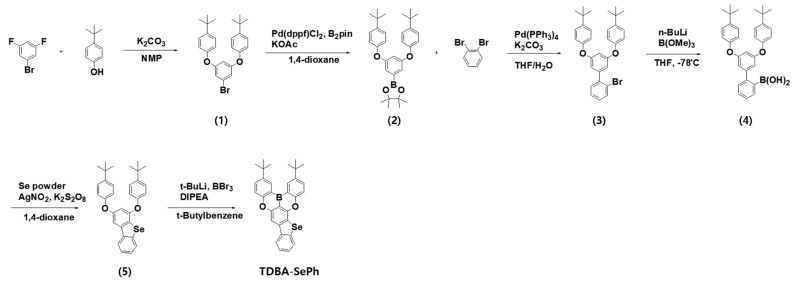
Synthetic route of the newly synthesized compound.

**Figure 1:**
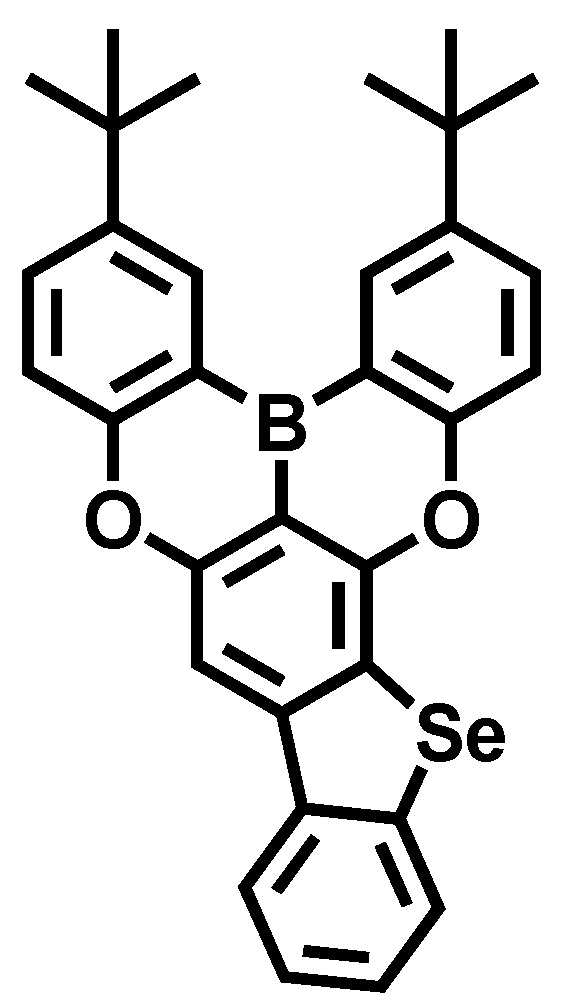
Chemical structure of the synthesized TDBA-based host material.

Scheme 1 illustrates the synthetic routes for the synthesized TDBA-SePh. In this study, the OLED host material was designed based on the TDBA framework, incorporating a Se atom into the structure to investigate the effects of heteroatom substitution (Figure 1). The TDBA framework is known for its MR-TADF characteristics and excellent electron-transporting properties, making it a suitable host material for achieving high-efficiency OLEDs [29,30]. In this study, we aimed to improve the TDBA structure by incorporating a Se atom with donating properties [27,31]. Selenium was incorporated into the host matrix to achieve the following effects. The selenium atom can enhance SOC through the heavy-atom effect, thereby facilitating the RISC process. Einzinger et al. and Huang et al. demonstrated that embedding a heavy atom like bromine within the host structure led to a reduction in the dopant’s triplet exciton lifetime, thereby improving efficiency roll-off characteristics [32,33]. Our group has also previously reported that the incorporation of silicon and germanium atoms led to an increase in the RISC rate and resulted in excellent device performance [29,34]. In this study, selenium atoms were introduced to exploit a similar heavy-atom effect. Although the use of Se in MR-TADF systems has not been extensively studied, it is anticipated to enhance SOC through its intrinsic heavy-atom characteristics. The Se atom was introduced at a position connecting one side of the TDBA core and the phenyl group, forming a fused structure. This molecular design is expected to maintain the rigidity of TDBA and effectively minimize non-radiative decay [35]. The detailed synthetic procedure is summarized in Scheme 1. The reaction intermediates, compounds (**2**), (**3**), and (**4**), were synthesized via borylation and Suzuki coupling reactions. The reaction intermediate compound (**5**) was synthesized through silver-catalyzed radical seleno cyclization under oxidative conditions. The final compound was obtained through a borylate cyclization reaction. The structure of the synthesized compounds was confirmed using mass spectrometry analysis and NMR spectroscopy (Figures S1–S6).

**Figure 2:**
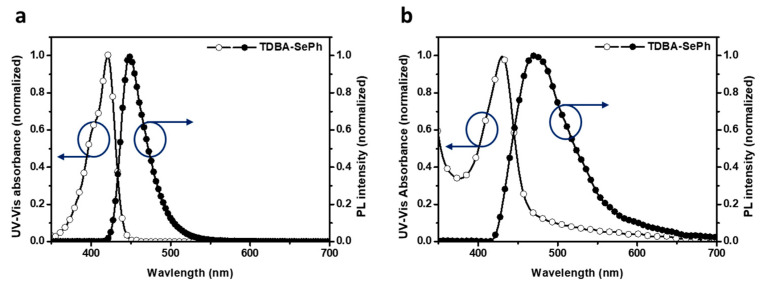
UV–vis absorption and PL spectra of TDBA-SePh in (**a**) solution state (toluene, 1 × 10^−5^ M) and (**b**) film state (thickness: 50 nm).

To evaluate the photophysical properties of TDBA-SePh, UV-Vis absorption, and photoluminescence (PL) spectra were measured in both solution and film states (Figure 2). Table 1 summarizes the photophysical characteristics of TDBA-SePh. The maximum absorption wavelength was observed at 420 nm in solution and 430 nm in the film state, indicating a 10 nm red shift due to intermolecular interactions in the solid state. PL spectrum analysis showed that the PL maximum (PL_max_) in the solution phase appeared at 447 nm with an FWHM of 35 nm, whereas in the thin-film state, the PL_max_ shifted to 468 nm with an increased FWHM of 78 nm. This corresponds to a 38 nm red shift in PL_max_ and a 43 nm broadening of the FWHM in the film, attributed to stronger intermolecular interactions and aggregation effects in the solid phase. The observed effect is due to the planar configuration of TDBA, in which the para-positioned phenyl group is fused with the TDBA core, leading to increased structural rigidity. This enhanced rigidity appears to influence molecular aggregation and π-π stacking interactions in the solid phase. The photoluminescence quantum yield (PLQY) was determined to be 14.0% in solution and 8.6% in the thin-film state. When TDBA-SePh was doped at a concentration of 3 wt% into the mCBP host matrix, the resulting film exhibited a PLQY of 37%. To evaluate the effect of selenium incorporation on the ΔE_ST_, low-temperature photoluminescence (LTPL) spectra were recorded for both TDBA and TDBA-SePh (Figure S7). The S_1_ and T_1_ energy levels of TDBA-SePh were found to be 2.90 eV and 2.66 eV, respectively, while those of TDBA were 3.27 eV and 3.03 eV. Although both energy levels were lowered by 0.37 eV upon selenium incorporation, the ΔE_ST_ remained unchanged at 0.24 eV for both materials.

HOMO levels for both materials were obtained through AC-2 analysis, and the corresponding LUMO energies were derived by combining the measured HOMO values with the optical band gaps estimated from UV–Vis absorption spectra. The optical band gap was obtained from the (*hν*) vs. (*αhν*)^2^ plot, where the absorbance edge was identified. In this expression, α represents absorbance, h denotes Planck’s constant, and ν stands for the frequency of light. TDBA-SePh exhibited HOMO and LUMO levels of −5.38 eV and −2.64 eV yielding an optical band gap of 2.74 eV. In comparison, the previously reported TDBA material from our group showed energy levels of −6.02 eV (HOMO) and −2.87 eV (LUMO), with a band gap of 3.15 eV [29]. The introduction of selenium into the TDBA backbone led to an elevation of the HOMO level by 0.64 eV and the LUMO level by 0.23 eV compared to the original TDBA. Compared to TDBA, TDBA-SePh exhibited a reduced HOMO–LUMO band gap by approximately 0.41 eV. As a result, the incorporation of selenium into the TDBA structure led to elevated HOMO and LUMO energy levels, accompanied by a narrowed band gap, which can be attributed to the electron-rich nature of selenium with its high atomic number. This observation is consistent with the findings of Jin et al. [31], who reported that the introduction of selenium atoms into organic molecules improves electronic properties due to their electron-donating characteristics.

### 3.2. Theoretical Calculation

**Figure 3:**
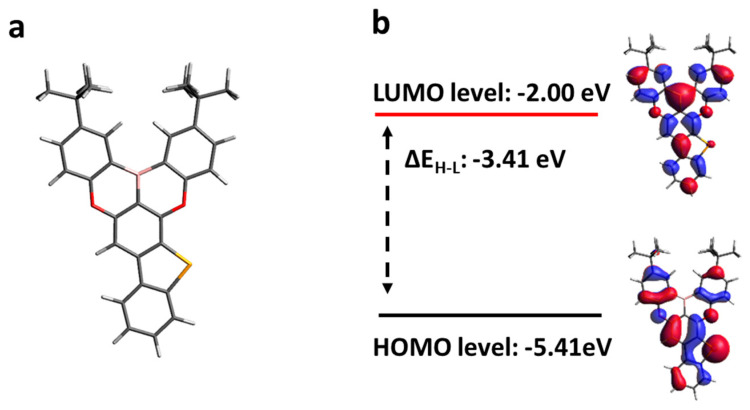
(**a**) Optimized structures and (**b**) electron density distributions of HOMO/LUMO energy levels calculated using B3LYP-D3/def2-TZVPP with ORCA.

The optimized molecular structures were investigated through density functional theory (DFT) calculations using the ORCA package, using the B3LYP-D3 functional with the def2-TZVPP basis set. As depicted in Figure 3a, the TDBA core and phenyl unit are fused through the Se atom, leading to a highly planar molecular conformation. This structural feature corresponds well with the broad FWHM of 78 nm observed in the thin-film state of TDBA-SePh. To further explore the electronic characteristics of TDBA-SePh, density functional theory (DFT) and time-dependent (TD)-DFT computations were performed. According to the theoretical calculations, the HOMO and LUMO energy levels were determined as −5.41 eV and −2.00 eV. The electron density distribution of the HOMO and LUMO orbitals was observed to be delocalized across both the TDBA core and the phenyl group (Figure 3b). This suggests that the incorporation of the Se atom, which forms a fused structure, extends the π-conjugation, allowing electron distribution to reach the para-positioned phenyl group. The HOMO is predominantly localized on the carbon atoms at the meta positions of the oxygen and boron centers, whereas the LUMO is mainly concentrated on the boron atom and the ortho- and para-positioned carbon atoms. This specific alternating electron density distribution in the HOMO and LUMO orbitals provides evidence of the MR effect in this molecular system [36,37]. Furthermore, theoretical calculations were performed to assess the S_1_ and T_1_ energy states, and SOC matrix elements were obtained using the ORCA computational program with the same basis set. The calculated SOC values of TDBA-SePh for S_1_ → T_1_ and S_1_ → T_2_ transitions were 0.15 cm^−1^ and 0.47 cm^−1^ (Figure S8). In comparison, previously reported SOC values for TDBA were 0.01 cm^−1^ for S_1_ → T_1_ and 0.17 cm^−1^ for S_1_ → T_2_. These findings indicate that the incorporation of Se into TDBA-SePh effectively enhances SOC interaction, leading to improved intersystem crossing efficiency. Transient photoluminescence (TRPL) measurements were performed on both TDBA and TDBA-SePh in solution (Figure S9). For TDBA-SePh, a distinct delayed fluorescence lifetime was observed, with a value of 0.38 μs. In contrast, no delayed fluorescence was detected in the case of TDBA. These results suggest that the selenium atoms in TDBA-SePh enhance SOC and enable the RISC process, leading to the observed delayed fluorescence lifetime. As a result, TDBA-SePh exhibits both MR-TADF characteristics and enhanced SOC properties, making it a promising host material capable of achieving high efficiency when applied in OLEDs. However, due to its molecular structure, the dominant luminescence mechanism is anticipated to be RISC from T_1_ to S_1_ [29,34].

### 3.3. Thermal Properties

**Figure 4:**
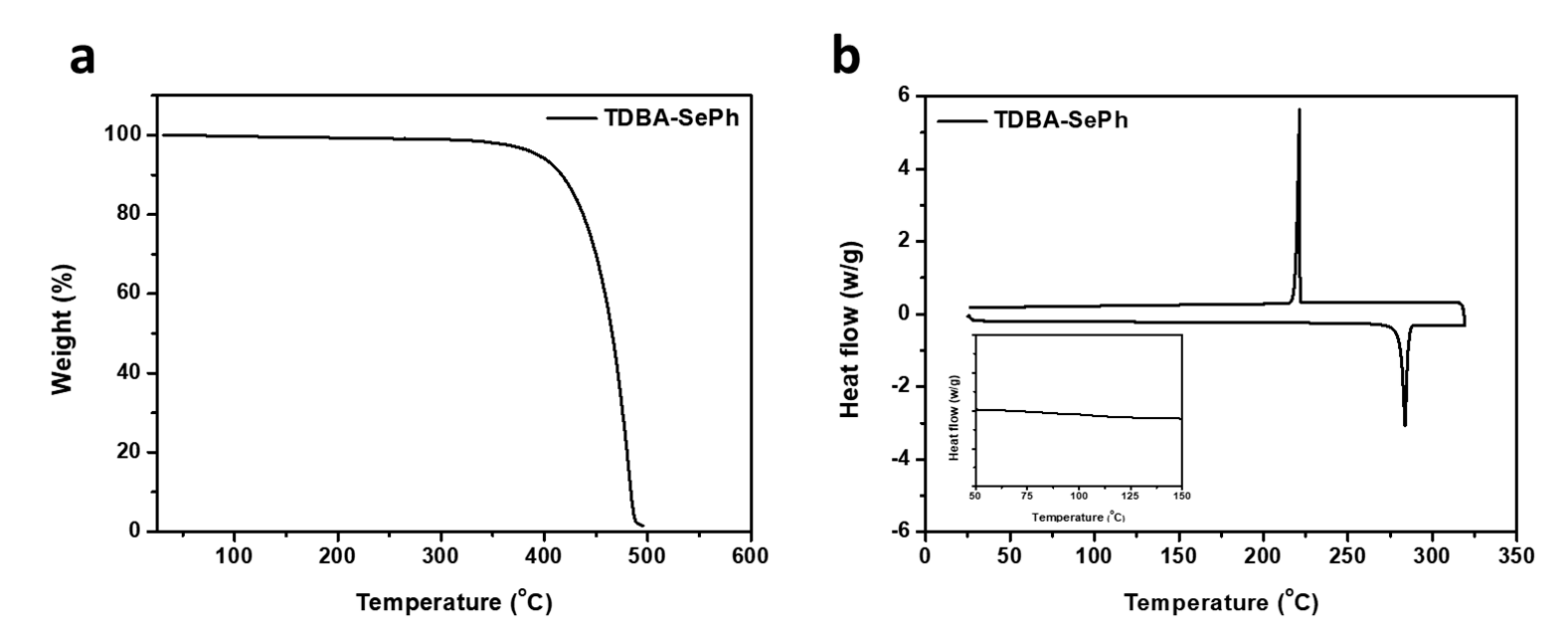
(**a**) TGA thermogram of the sample including an additional isothermal hold at 200 °C for 20 min to evaluate thermal stability; (**b**) DSC curve with an expanded view of the glass transition (T_g_) region shown in the inset.

In this study, thermogravimetric analysis (TGA) and differential scanning calorimetry (DSC) measurements were conducted to evaluate the thermal stability of the synthesized selenium-based OLED host material (Figure 4). To further evaluate the thermal stability of TDBA-SePh, the sample was heated from 30 °C to 200 °C at a rate of 10 °C/min and held isothermally at 200 °C for 20 min. Subsequently, the temperature was increased to 500 °C at the same heating rate (10 °C/min) to complete the TGA measurement. The isothermal hold at 200 °C for 20 min was employed because this method is commonly used in industrial settings to evaluate the thermal stability of OLED materials. Under these conditions, the decomposition temperature (T_d_, corresponding to 5% weight loss) was determined to be 393 °C. Furthermore, the mass change graph during the 20-min isothermal period at 200 °C (Figure S10) showed that the material retained nearly constant weight, indicating that the thermal stability of TDBA-SePh is well maintained even under prolonged high-temperature conditions. When compared to the T_d_ of 184 °C for the TDBA framework, this result confirms the enhanced thermal stability of the synthesized compound [29]. The high decomposition temperature is a crucial factor in ensuring long-term device stability and thermal resistance during high-temperature processing. Furthermore, DSC analysis was performed to determine the glass transition temperature (T_g_), crystallization temperature (T_c_), and melting temperature (T_m_). The measured values were 102 °C for T_g_, 221 °C for T_c_, and 273 °C for T_m_, indicating the thermal stability and processability of the synthesized material. The excellent thermal properties of this compound ensure structural stability even under the thermal stress generated during device operation. These thermal characteristics are expected to contribute to the stable performance of the material when applied as an emission layer in OLED devices.

### 3.4. Electroluminescence Properties

**Figure 5:**
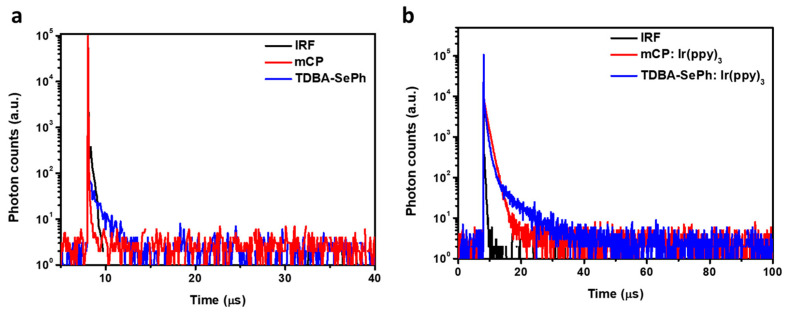
Transient photoluminescence decay spectra of (**a**) neat films and (**b**) doped films (IRF: instrument response function).

Considering that the T_1_ energy level of TDBA-SePh is 2.66 eV, it was evaluated as a host material for green and red phosphorescent dopants. The green device employed tris(2-phenylpyridine)iridium (Ir(ppy)_3_) as the dopant, while the red device utilized bis(1-phenylisoquinoline)(acetylacetonate)iridium(III) (Ir(piq)_2_acac) as the emissive dopant. A device incorporating TDBA as the host was initially planned for comparison. However, preliminary experiments revealed that TDBA alone exhibits insufficient thermal stability and suboptimal film morphology, making it unsuitable for direct OLED fabrication and performance evaluation [29]. Consequently, the performance of TDBA-SePh-based devices was compared with OLEDs employing mCP, a well-established and widely used host material. To evaluate how efficiently energy is transferred from the host material to Ir(ppy)_3_, Stern–Volmer experiments were carried out, with the results summarized in Figure S11 and Table S4. The quenching rate constants (*k*_2_) for mCP and TDBA-SePh were determined to be 1.25 × 10^7^ s^−1^ and 1.49 × 10^7^ s^−1^ suggesting that energy transfer occurs slightly more efficiently in TDBA-SePh compared to mCP. The PLQY values of 10 wt% doped films were measured as 28% for mCP and 43% for TDBA-SePh, indicating a higher quantum yield for TDBA-SePh. Furthermore, transient PL measurements were performed on neat films of mCP and TDBA-SePh to observe delayed fluorescence (Figure 5a and Table S1). No delayed fluorescence was detected in mCP, whereas in TDBA-SePh neat films, a delayed lifetime of 1.89 μs and a reverse intersystem crossing (*k*_RISC_) rate constant of 3.91 × 10^4^ s^−1^ were observed. Additionally, to investigate the doped film state properties of mCP and TDBA-SePh, doped films were fabricated using Ir(ppy)_3_ as the dopant, and transient PL measurements were performed. The RISC rate calculated from the PLQY values was 1.43 × 10^4^ s^−1^ for mCP and 6.53 × 10^4^ s^−1^ for TDBA-SePh (Figure 5b and Table S2). These results suggest that TDBA-SePh can serve as a superior host material compared to mCP, owing to its efficient energy transfer to the dopant, higher fluorescence efficiency, and enhanced RISC rate.

A non-doped OLED device was fabricated and evaluated using TDBA-SePh as the emitting layer. As shown in Figure S12, the non-doped device was constructed with the following layer structure: ITO/4,4′,4″-Tris(N-(2-naphthyl)-N-phenyl-amino)-triphenylamine (2-TNATA) (60 nm)/N,N′-bis(naphthalene-1-yl)-N,N′-bis(phenyl)benzidine (NPB) (15 nm)/TDBA-SePh (35 nm)/TPBi (30 nm)/LiF (1 nm)/Al (200 nm). In this configuration, 2-TNATA, NPB, and TPBi were employed as the hole injection layer (HIL), hole transport layer (HTL), and electron transport layer (ETL), respectively. The energy level diagram and chemical structures of the materials utilized in the non-doped OLED devices are depicted in Figure S12. Figure S13a–d displays the (current density) J-(voltage) V-(luminance) L characteristics, (current efficiency) CE-J plots, (external quantum efficiency) EQE-J curves, and EL spectra of the fabricated devices. A detailed summary of the EL characteristics of the non-doped OLED can be found in Table S5. The non-doped OLED device exhibited a typical J-V-L curve, with a turn-on voltage (V_on_) of approximately 5.5 V. As shown in Figure S13d, the EL spectrum revealed an EL maximum peak at 474 nm, which closely matched the PL characteristics observed in the thin-film state. The non-doped device emitted light with Commission Internationale de l’Éclairage (CIE) coordinates chromaticity coordinates of (0.162, 0.278) and demonstrated a CE of 0.65 cd/A and an EQE of 0.41%. To evaluate the electroluminescent potential of TDBA-SePh as a dopant, an OLED device was fabricated using mCBP as the host material with 3 wt% TDBA-SePh doping. Except for the emitting layer, the device configuration was identical to that shown in Figure S12. The doped device exhibited a turn-on V_on_ of 3.95 V, a CE of 1.3 cd/A, and an(EQE of 2.3%. The EL spectrum showed an FWHM of 35 nm, and the CIE coordinates were measured to be (0.149, and 0.056) (Figure S14 and Table S6). Compared to the non-doped device, the doped device demonstrated enhanced performance, which is consistent with the significant increase in PLQY from 8.6% (neat film) to 37% in the mCBP: 3 wt% TDBA-SePh doped film. Figure S12b,c depict the energy level diagrams and molecular structures of the materials employed in the doped OLED devices. The detailed device architecture used in the experiments is as follows. Device 1: ITO/NPB (40 nm)/TCTA (15 nm)/mCP (15 nm)/mCP: 10 wt% Ir(ppy)_3_ (20 nm)/TmPyPB (40 nm)/LiF (1 nm)/Al (200 nm), Device 2: ITO/NPB (40 nm)/TCTA (15 nm)/mCP (15 nm)/TDBA-SePh: 10 wt% Ir(ppy)_3_ (20 nm)/TmPyPB (40 nm)/LiF (1 nm)/Al (200 nm), Device 3: ITO/NPB (40 nm)/TCTA (15 nm)/mCP (15 nm)/mCP: 10 wt% Ir(piq)_2_acac (20 nm)/TmPyPB (40 nm)/LiF (1 nm)/Al (200 nm), Device 4: ITO/NPB (40 nm)/TCTA (15 nm)/mCP (15 nm)/TDBA-SePh: 10 wt% Ir(piq)_2_acac (20 nm)/TmPyPB (40 nm)/LiF (1 nm)/Al (200 nm). The device performance of the doped OLEDs is summarized in Table 2.

**Figure 6:**
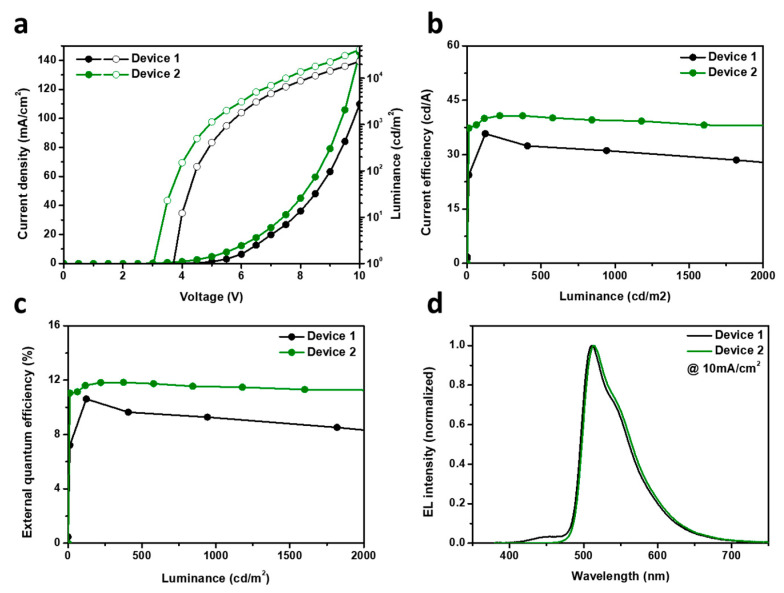
EL characteristics of doped devices using TDBA-SePh and Ir(ppy)_3_: (**a**) J-V-L curve, (**b**) luminance efficiency versus current density, (**c**) external quantum efficiency versus current density, and (**d**) EL spectrum of OLED doped devices at 10 mA/cm^2^.

Evaluation of the EL characteristics using a green dopant revealed that the device utilizing TDBA-SePh as the host exhibited a lower driving voltage and higher current efficiency compared to the conventional mCP-based device (Figure 6). In Device 1, the mCP-based host device exhibited a Von of 3.71 V, with CE and EQE of 25.9 cd/A and 7.8%, respectively. The TDBA-SePh-based host device showed a V_on_ of 3.05 V, with CE at 33.0 cd/A and EQE at 9.3%. Furthermore, the FWHM values for Device 1 and Device 2 were 67 nm and 65 nm, and the CIE (x, y) coordinates were (0.293, 0.601) and (0.309, 0.621), respectively.

**Figure 7:**
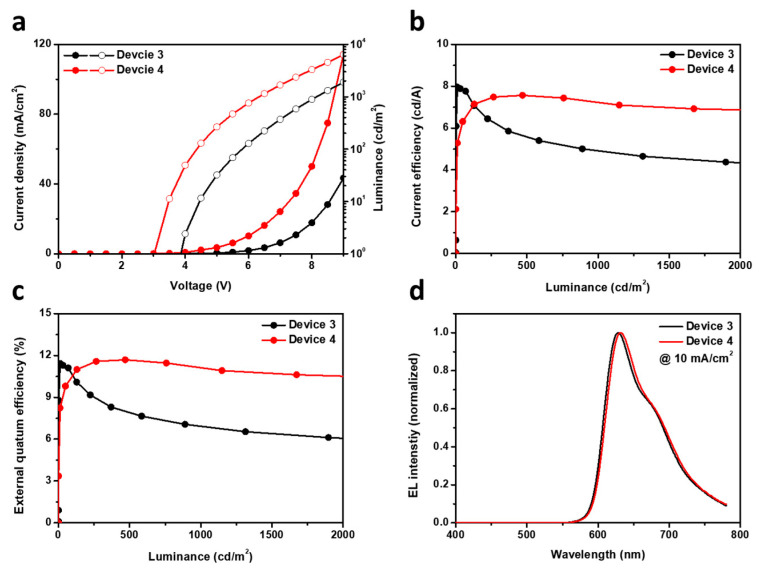
EL characteristics of doped devices using TDBA-SePh and Ir(piq)_2_acac: (**a**) J–V–L curves, (**b**) current efficiency versus current density, (**c**) external quantum efficiency versus current density, and (**d**) EL spectrum of the doped OLED devices at 10 mA/cm^2^.

Additionally, the EL characteristics were evaluated using a red dopant. In the case of the red-doped device, the TDBA-SePh-based host outperformed the conventional mCP-based device, showing a lower driving voltage and higher current efficiency (Figure 7). For Device 3, the mCP-based host device had a V_on_ of 3.86 V, with CE and EQE measured at 5.5 cd/A and 7.8%, respectively. The TDBA-SePh-based host device (Device 4) showed a V_on_ of 3.01 V, with CE of 7.4 cd/A and EQE of 11.5%. The FWHM values for Device 3 and Device 4 were measured at 85 nm and 84 nm and both devices exhibited similar CIE (x, y) values, (0.682, 0.317) and (0.687, 0.313), indicating similar EL spectra. In addition, the EQE roll-off of Device 1 and Device 2 was compared. As shown in Figure 6c, at maximum luminance/500 cd m^−2^/1000 cd m^−2^, Device 1 exhibited 10.6%/9.6%/9.2% and Device 2 showed 11.8%/11.7%/11.5%. As shown in Figure 7c, Device 3, and Device 4, the EQE values at maximum luminance/500 cd m^−2^/1000 cd m^−2^ were 10.9%/7.9%/6.9% and 11.7%/11.6%/11.2%, respectively. When TDBA-SePh was used as the host for green and red dopants, the roll-off values were found to be around 2.5% and 4.3%, demonstrating outstanding roll-off characteristics. Although the current device using the TDBA-SePh host exhibited higher EL efficiency than the mCP-based device, a previous study by Gao et al. in 2014 reported a CE of 65.2 cd/A in a green phosphorescent OLED using a CBP host doped with 10 wt% Ir(ppy)_3_, which surpasses the 33.0 cd/A observed in our study [38]. However, in that case, the device suffered from a significant efficiency roll-off of approximately 10%, indicating a critical limitation in device performance. Such roll-off behavior is presumed to originate from triplet-triplet interactions, which not only reduce efficiency but also negatively affect device lifetime. In contrast, the Hatakeyama group in 2015 demonstrated that phosphorescent OLEDs using a 5,9-dioxa-13b-boranaphtho[3,2,1-de]anthracene (DOBNA) derivative as the host achieved superior performance, with an EQE_max_ of 20.1% and a device lifetime of 1000 h, outperforming the CBP-based counterpart that showed 17.6% EQE_max_ and lasted only 80 h at a luminance of 2000 cd·m^−2^ [39]. Accordingly, future studies focusing on the optimization of device structures and fabrication conditions are expected to further improve efficiency. MR-TADF host materials show great potential as next-generation alternatives to conventional hosts, owing to their advantages in EL efficiency, mitigation of efficiency roll-off, and enhanced operational stability. The TDBA-SePh host exhibited remarkably suppressed efficiency roll-off values of 2.5% with Ir(ppy)_3_ and 4.3% with Ir(piq)_2_acac dopants. MR-TADF host materials possess a small ΔE_ST_, which facilitates RISC through thermal activation, enabling the upconversion of nonradiative triplet excitons into radiative singlet excitons. Consequently, MR-TADF host materials can lower the triplet exciton density in the emissive layer, which helps suppress triplet-induced degradation and enhance efficiency roll-off performance [40]. These results suggest that, based on the heteroatom effect, the RISC process occurs more rapidly, leading to a reduction in triplet-level stagnation and a decrease in the population of triplet excitons. This results in not only a reduction in efficiency roll-off but also an improvement in EL performance. As a result, the TDBA-SePh suggested in this study demonstrates significant potential as a host material for next-generation green and red phosphorescent OLEDs, with excellent EL properties, including low driving voltage and high current efficiency.

## 4. Conclusions

In this study, we successfully designed and synthesized a new Se-contained MR-TADF host material, TDBA-SePh, for green and red PhOLED applications. By integrating Se into the DOBNA-based MR-TADF host structure, the SOC was enhanced, and the RISC process was accelerated, showing a RISC rate of 3.91 × 10^4^ s^−1^. This improvement is attributed to the effective utilization of triplet excitons, which contributed to enhanced device performance. The TDBA-SePh exhibited excellent thermal stability, with T_d_ and T_g_ values of 393 °C and 102 °C. When used as the host material in PhOLED devices, the TDBA-SePh-based green and red PhOLEDs demonstrated superior driving voltage and device efficiency compared to conventional mCP-based devices. Additionally, at a high luminance of 1000 cd m^−2^, the roll-off values for the green and red devices were 2.5% and 4.3% indicating low roll-off and high device efficiency. These findings suggest that the heteroatom effect of selenium in the MR-TADF host leads to an accelerated RISC process, reducing triplet level stagnation and lowering the triplet exciton population, which in turn improves device performance. This research proposes a new strategy for designing host materials for next-generation green and red phosphorescent OLEDs, enhancing the performance of PhOLEDs.

## Figures and Tables

**Table 1:** Photophysical properties of TDBA-SePh.

	Solution ^a^				Film ^b^								
	λ_Abs_ (nm)	λ_PL_ (nm)	FWHM ^c^ (nm)	ϕ_F_ (%)	λ_Abs_ (nm)	λ_PL_ (nm)	FWHM (nm)	ϕ_F_ (%)	E_S_/E_T_/ΔE_ST_ (eV)	Τ_d_ (μs)	HOMO ^d^ (eV)	LUMO (eV)	Band Gap (eV)
TDBA-SePh	420	447	35	14.0	430	468	78	8.6	2.90/2.66/0.24	1.42	−5.38	−2.64	2.74

^a^ Toluene solution (1.0 × 10^−5^ M). ^b^ Film thickness is 50 nm on the glass. ^c^ Full width at half maximum of PL. ^d^ Ultraviolet photoelectron spectroscopy (Riken-Keiki, AC-2, Tokyo, Japan).

**Table 2:** EL performances of the fabricated OLED doped devices at 10 mA/cm^2^.

Devices	V_on_ ^a^ (V)	LE (cd/A)	Luminance (cd/m^2^)	EQE (%)	CIE (x, y)	EL_max_ (nm)	FWHM (nm)
Device 1	3.71	25.9	2495	7.8	(0.293, 0.601)	511	64
Device 2	3.05	33.0	3100	9.3	(0.309, 0.621)	513	66
Device 3	3.86	5.5	538	7.8	(0.682, 0.317)	629	85
Device 4	3.01	7.4	759	11.5	(0.687, 0.313)	631	84

^a^ Turn-on voltage at 1 cd m^−2^.

## Data Availability

The original contributions presented in this study are included in the article/Supplementary Materials. Further inquiries can be directed to the corresponding author.

## Supplementary Materials

The following supporting information can be downloaded at: https://www.mdpi.com/article/10.3390/ma18092040/s1, Figure S1. ^1^H-NMR spectrum of compound (**1**).; Figure S2. ^1^H-NMR spectrum of compound (**2**).; Figure S3. ^1^H-NMR spectrum of compound (**3**).; Figure S4. ^1^H-NMR spectrum of compound (**4**).; Figure S5. ^1^H-NMR spectrum of compound (**5**).; Figure S6. ^1^H-NMR spectrum of TDBA-SePh.; Figure S7. Photoluminescence (black) at room temperature, low-temperature photoluminescence with delay (Red) at 77K spectra of solution state. (a) TDBA and (b) TDBA-SePh.; Figure S8. Isosurface of HOMO and LUMO composing S_0_→S_1_ transition (isovalue = 0.02) with representative electronic transition energies with SOC values of TDBA-SePh. TD-B3LYP calculation was conducted at the level of 6–31G(d,p).; Figure S9. Transient photoluminescence decay spectra of TDBA and TDBA-SePh in solution state (IRF: instrument response function). Figure S10. Mass change over 20 min under the isothermal condition at 200 °C.; Figure S11. Stern-Volmer plots: (a) mCP, (b) TDBA-SePh.; Figure S12. Energy level diagram of (a) non-doped OLED device, (b) doped OLED devices, and (c) molecular structures used in each layer; Figure S13. EL characteristics of non-doped device using TDBA-SePh: (a) J-V-L curve, (b) luminance efficiency versus current density, (c) external quantum efficiency versus current density, and (d) EL spectrum of OLED doped devices at 10 mA/cm^2^.; Figure S14. EL characteristics of doped device TDBA-SePh as a dopant in emitting layer: (a) J-V-L curve, (b) luminance efficiency versus current density, (c) external quantum efficiency versus current density, and (d) EL spectrum of OLED doped devices at 10 mA/cm^2^.; Table S1. Rate constant for TDBA-SePh (non-doped film) at room temperature.; Table S2. The rate constant for doped film using mCP and TDBA-SePh at room temperature. Table S3.; Thermal properties of the synthesized materials.; Table S4. The rate constant of energy transfer between the host and dopant is based on the Stern-Volmer equation.; Table S5. EL performance of the non-doped OLED device at 10 mA/cm^2^.; Table S6. EL performance of the doped OLED device at 10 mA/cm^2^.
