# Supplementary material for: Selenium-Containing Multi-Resonance Thermally Activated Delayed Fluorescence Host Material for Green and Red Phosphorescent OLEDs
Source: Materials (Basel). 2025 Apr 29;18(9):2040. doi: 10.3390/ma18092040 (PMC12072477; doi:10.3390/ma18092040)
Supplement: Supplementary file 1 [file materials-18-02040-s001.zip › materials-3578837-supplementary.pdf]

materials

Supplementary Materials

Selenium-Containing Multi-Resonance Thermally Activated Delayed Fluorescence Host Material for Green and Red Phosphorescent OLEDs

Hyukmin Kwon †, Seokwoo Kang †, Sangwook Park, Saeyoung Oh, Sang-Tae Kim, Kiho Lee, Hayoon Lee and Jongwook Park *

Integrated Engineering, Department of Chemical Engineering, Kyung Hee University, Yongin-si 17104, Republic of Korea
* Correspondence: jongpark@khu.ac.kr; Tel.: +82-10-8759-8485
† This author equally contributed to this work.

General information

All reagents and solvents were purchased as reagent grade and used without further purification. Analytical thin-layer chromatography (TLC) was conducted using Merck60 F254 silica gel plates, and column chromatography was performed using Merck 60 silica gel (Burlington, MA, USA), (230–400 mesh). ^{1}H NMR spectra were recorded in chloroform-*d* ($CDCl_3$) and dimethylsulfoxide (DMSO-d_6) using a JNM-ECZ400S/L1 NMR spectrometer (JEOL, Tokyo, Japan) at ambient temperature. High-resolution mass spectrometry (HRMS) was performed using fast atom bombardment (FAB) using a JMS-700, 6890 Series mass spectrometer (JEOL, Tokyo, Japan). Optical UV–vis absorption spectra were recorded using a UV-1900i UV/Vis/NIR spectrometer (Shimadzu, Kyoto, Japan). Photoluminescence (PL) spectroscopy was conducted using a PerkinElmer LS55 luminescence spectrometer with an Xe flash lamp (PerkinElmer, Inc., Waltham, MA, USA). Absolute photoluminescence quantum yield (PLQY) measurements were carried out using a Hamamatsu Quantaurus-QY C11347 Absolute PL Quantum Yield spectrometer (Hamamatsu Photonics, Shizuoka-ken, Japan). Transient photoluminescence was measured using a Quantaurus-Tau fluorescence lifetime measurement system (Hamamatsu Photonics, Shizuoka-ken, Japan). Triplet energy (T_1) was determined by low-temperature photoluminescence (LTPL) measurement, performed using a FluoroMate FS-2 luminescence spectrophotometer (SCINCO, Seoul, Korea). Density functional theory (DFT) calculations for TDBA-SePh were performed using the B3LYP-D3 functional and the def2-TZVPP basis set using the ORCA program package (V 5.0.4). The glass transition temperature (T_g), melting temperature (T_m), and crystallization temperature (T_c) of the compounds were determined using differential scanning calorimetry (DSC) under a nitrogen atmosphere using a DSC 26 (TA Instruments, New Castle, DE, USA). Degradation temperatures (T_d) were measured based on a thermogravimetric analysis (TGA) using a SDT Q600 (TA Instruments, New Castle, DE, USA), with samples heated to 700 °C at a rate of 10 °C/min. The HOMO energy levels were determined using ultraviolet photoelectron yield spectroscopy (Riken Keiki AC-2, Nara, Japan), and the LUMO energy levels were derived from the HOMO energy levels and the bandgaps. Film samples of the synthesized compounds were deposited to a thickness of 50 nm at a deposition rate of 1 Å/s under a vacuum of 10^{-6} torr. For electroluminescence (EL) devices, all organic layers were deposited under the same vacuum conditions at a rate of 1 Å/s to cover an area of 4 mm^2. LiF and Al layers were deposited continuously under these vacuum conditions. The current–voltage–luminance (I–V–L) properties of the fabricated EL devices were measured using a Keithley 2400 electrometer (Tektronix, Cleveland, OH, USA), and the light intensities were measured using a Minolta CS-1000A (Konica Minolta, Tokyo, Japan). The devices were stored in a glovebox to maintain stability against moisture and air.

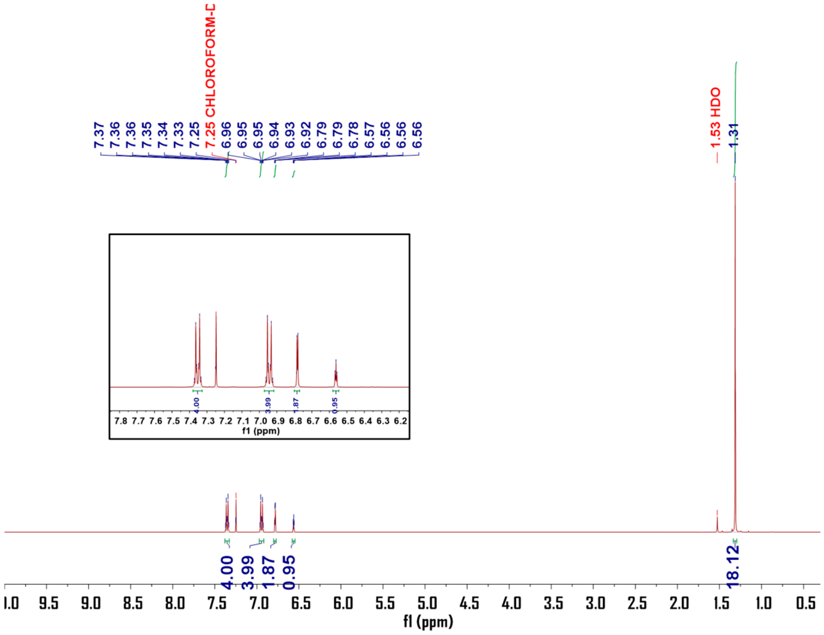

Figure S1. ^{1}H-NMR spectrum of compound (1).

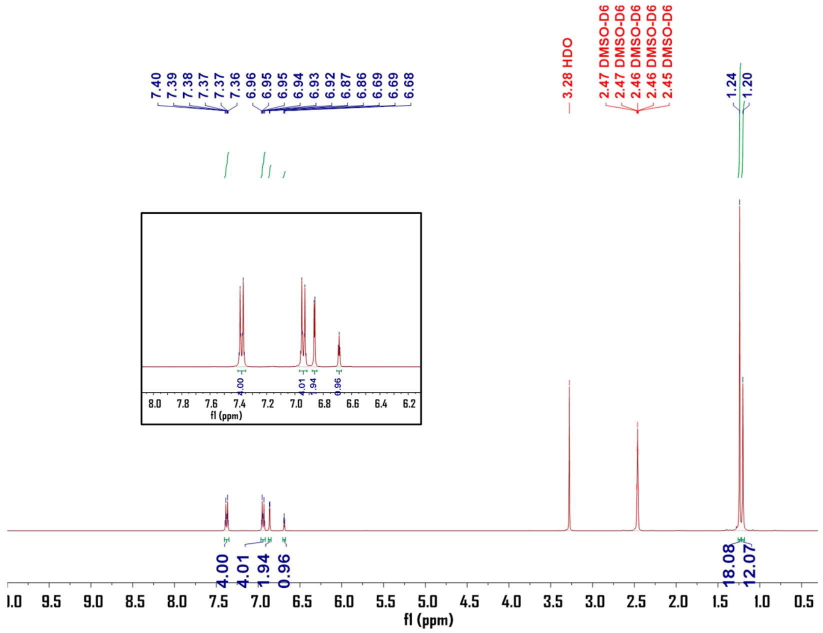

Figure S2. ^{1}H-NMR spectrum of compound (2).

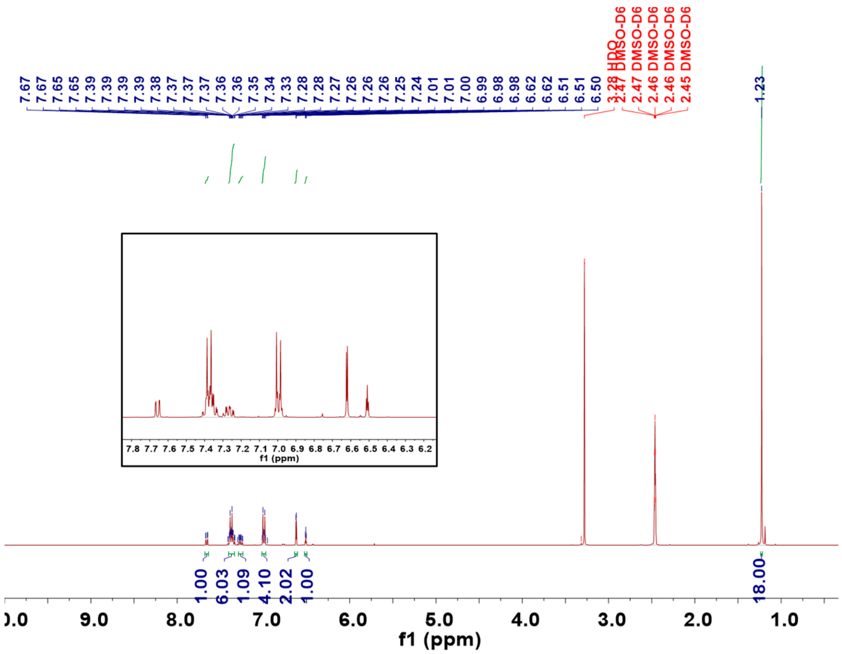

Figure S3. ^{1}H-NMR spectrum of compound (3).

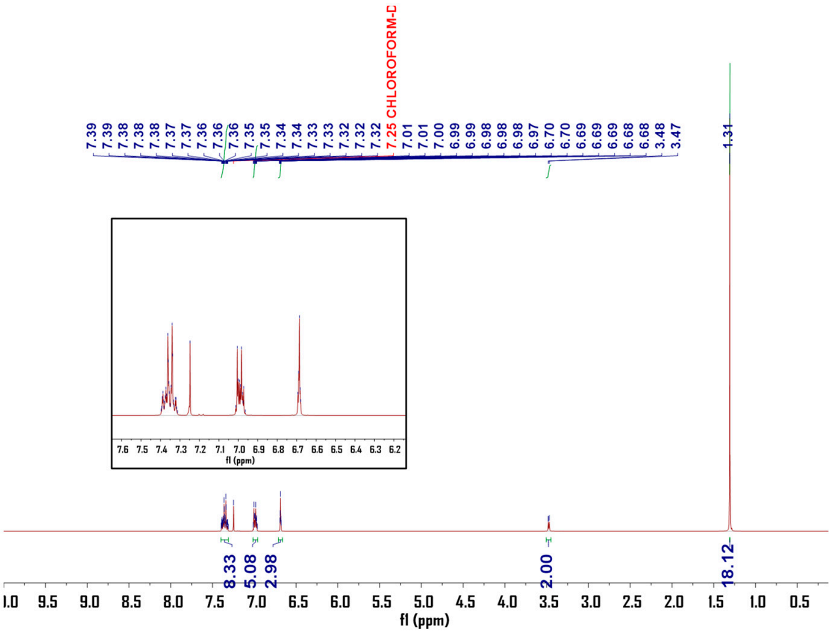

Figure S4. ^{1}H-NMR spectrum of compound (4).

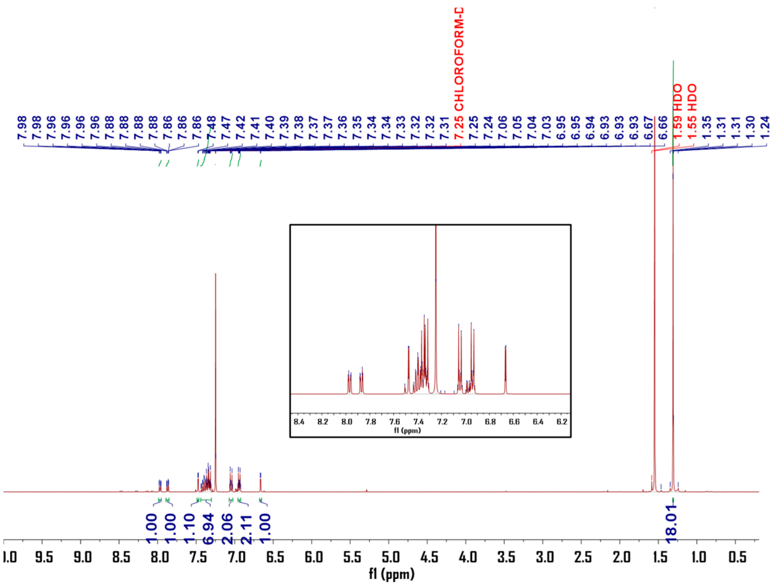

Figure S5. ^{1}H-NMR spectrum of compound (5).

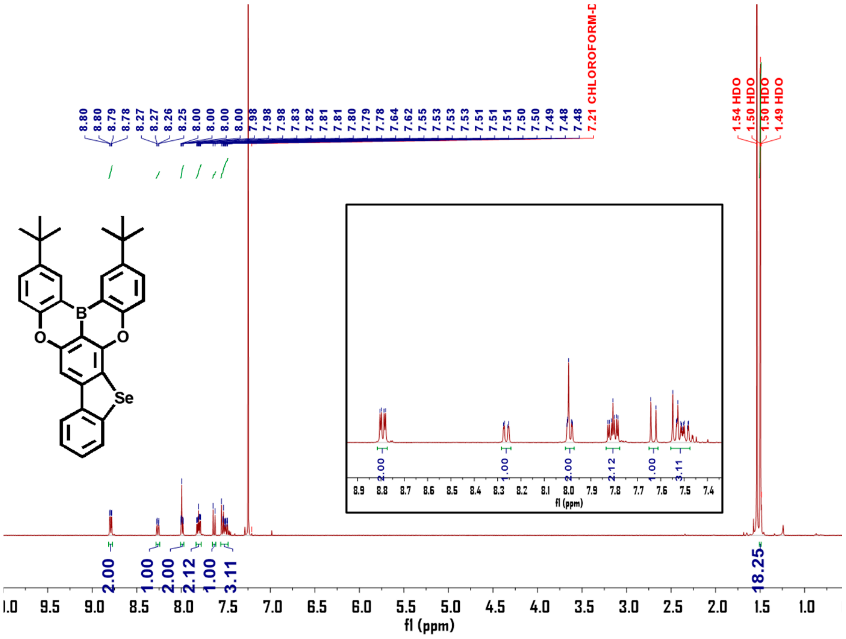

Figure S6. ^{1}H-NMR spectrum of TDBA-SePh.

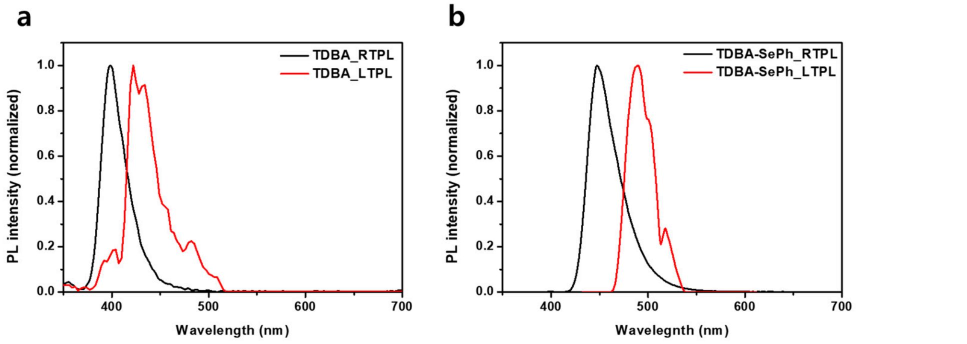

Figure S7. Photoluminescence (black) at room temperature, low temperature photoluminescence with delay (Red) at 77K spectra of solution state. (a) TDBA and (b) TDBA-SePh.

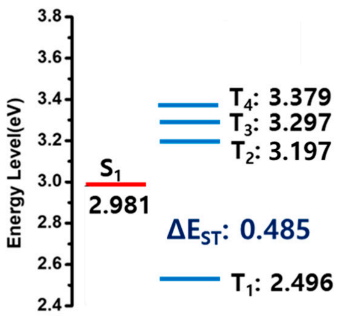

SOCC (cm^{-1})			
$\langle S_1 I \hat{H}SOC I T_1 \rangle$	$\langle S_1 I \hat{H}SOC I T_2 \rangle$	$\langle S_1 I \hat{H}SOC I T_3 \rangle$	$\langle S_1 I \hat{H}SOC I T_4 \rangle$
0.146	0.471	0.566	0.455

Figure S8. Isosurface of HOMO and LUMO composing $S_0 \rightarrow S_1$ transition (isovalue = 0.02) with representative electronic transition energies with SOC values of TDBA-SePh. TD-B3LYP calculation was conducted at the level of 6-31G(d,p).

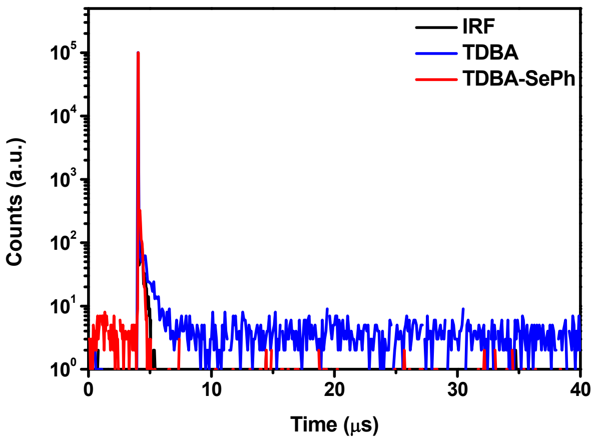

Figure S9. Transient photoluminescence decay spectra of TDBA and TDBA-SePh in solution state (IRF: instrument response function).

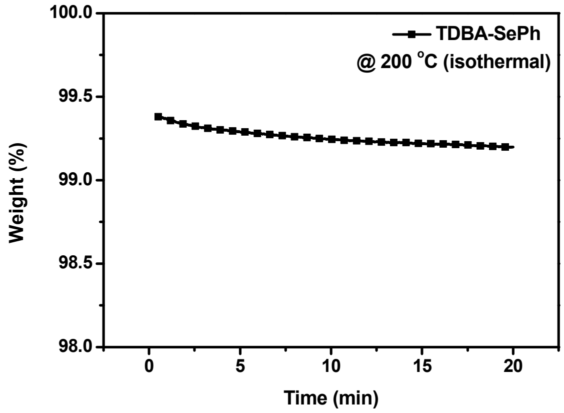

Figure S10. Mass change over 20 minutes under the isothermal condition at 200 °C.

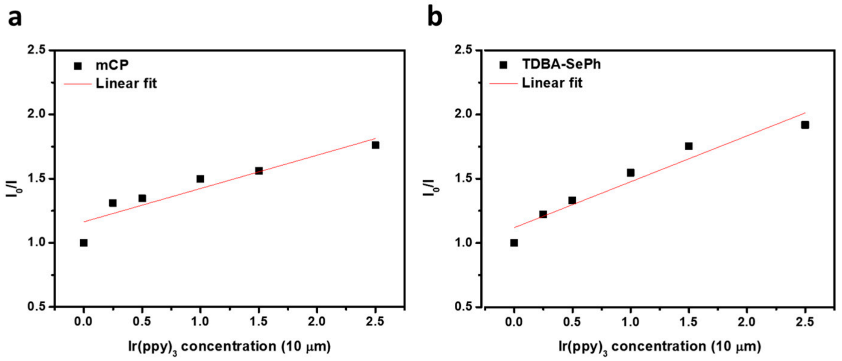

Figure S11. Stern-Volmer plots: (a) mCP, (b) TDBA-SePh.

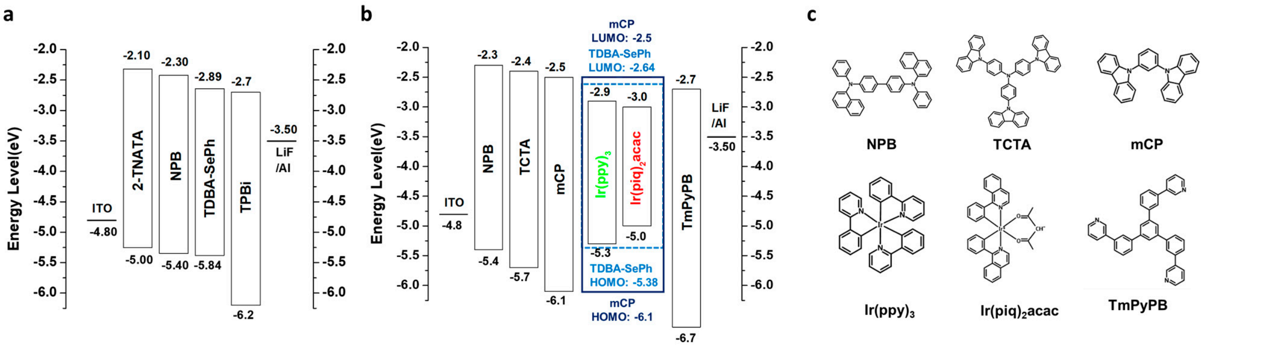

Figure S12. Energy level diagram of (a) non-doped OLED device and (b) doped OLED devices, and (c) molecular structures used in each layer.

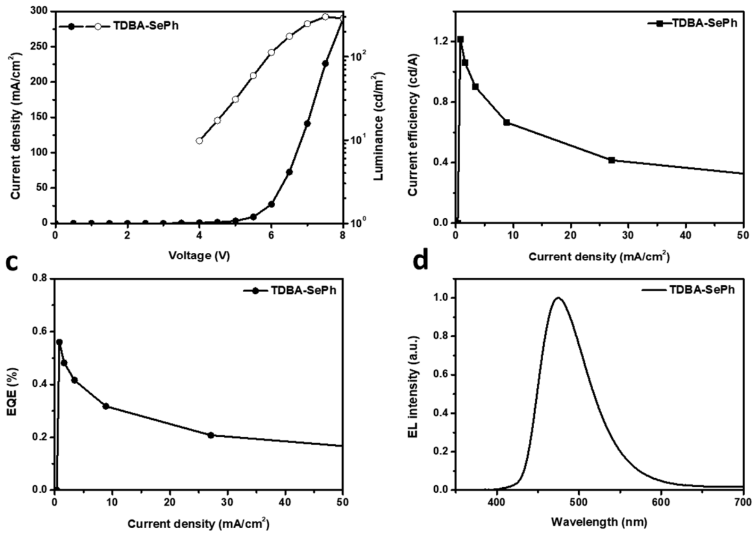

Figure S13. EL characteristics of non-doped device using TDBA-SePh: (a) J-V-L curve, (b) luminance efficiency versus current density, (c) external quantum efficiency versus current density, and (d) EL spectrum of OLED doped devices at 10 mA/cm^2.

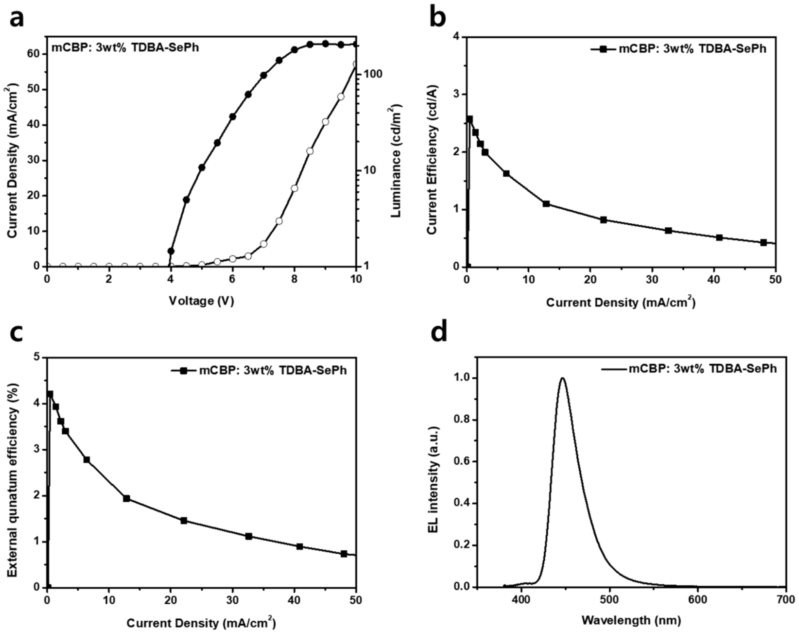

Figure S14. EL characteristics of doped device TDBA-SePh as dopant in emitting layer: (a) J-V-L curve, (b) luminance efficiency versus current density, (c) external quantum efficiency versus current density, and (d) EL spectrum of OLED doped devices at 10 mA/cm^2.

Table S1. Rate constant for TDBA-SePh (non-doped film) at room temperature.

**Materials**	**TDBA-SePh**
ϕ	0.086
ϕ_F	0.084
ϕ_{TADF}	0.002
τ_f (ns)	18.10
τ_{TADF} (μs)	1.89
K_F (x10^7)	0.47
K_{IC} (x10^7)	4.96
K_{ISC} (x10^7)	0.98
ϕ_{IC}	0.898
ϕ_{ISC}	0.02
K_{TADF} (x10^4)	4.54
K_{RISC} (x10^4)	3.91

Table S2. Rate constant for doped film using mCP and TDBA-SePh at room temperature.

	**mCP :Ir(ppy)3**	**TDBA-SePh :Ir(ppy)3**
ϕ	0.280	0.432
ϕ_F	0.253	0.379
ϕ_{TADF}	0.027	0.063
τ_f (ns)	22.3	60.7

τ_{TADF} (μs)	5.48	4.47
K_F (x10^7)	1.13	0.63
K_{IC} (x10^7)	2.92	0.83
K_{ISC} (x10^7)	0.43	0.19
ϕ_{IC}	0.65	0.50
ϕ_{ISC}	0.09	0.12
K_{TADF} (x10^4)	5.11	12.08
K_{RISC} (x10^4)	1.43	6.53

Table S3. Thermal properties of the synthesized materials.

Materials	T_g[a] (°C)	T_c[b] (°C)	T_m[c] (°C)	T_d[d] (°C)
TDBA-SePh	102	221	273	393

[a] Glass transition temperature. [b] Crystallization temperature [c] Melting temperature. [d] Decomposition temperature at 5% weight loss.

Table S4. Rate constant of energy transfer between host and dopant based on Stern-Volmer equation.

Materials	mCP	TDBA-SePh
Slope	0.26	0.37
K_q[a] (1/s)	1.25 x 10^7	1.49 x 10^7

[a]Energy transfer that occurs between host and dopant.

Table S5. EL performance of the non-doped OLED device at 10 mA/cm^2.

Devices	V_{on} [a] (V)	LE (cd/A)	Luminance (cd/m^2)	EQE (%)	CIE (x, y)	EL_{max} (nm)	FWHM (nm)
TDBA-SePh	5.51	0.65	60	0.31	(0.162, 0.278)	474	68

[a] Turn-on voltage at 1 cd m^{-2}.

Table S6. EL performance of the doped OLED device at 10 mA/cm^2.

Devices	V_{on} [a] (V)	LE (cd/A)	Luminance (cd/m^2)	EQE (%)	CIE (x, y)	EL_{max} (nm)	FWHM (nm)
mCBP: 3wt% TDBA-SePh	3.95	1.3	125	2.3	(0.149, 0.056)	447	35

[a] Turn-on voltage at 1 cd m^{-2}.
